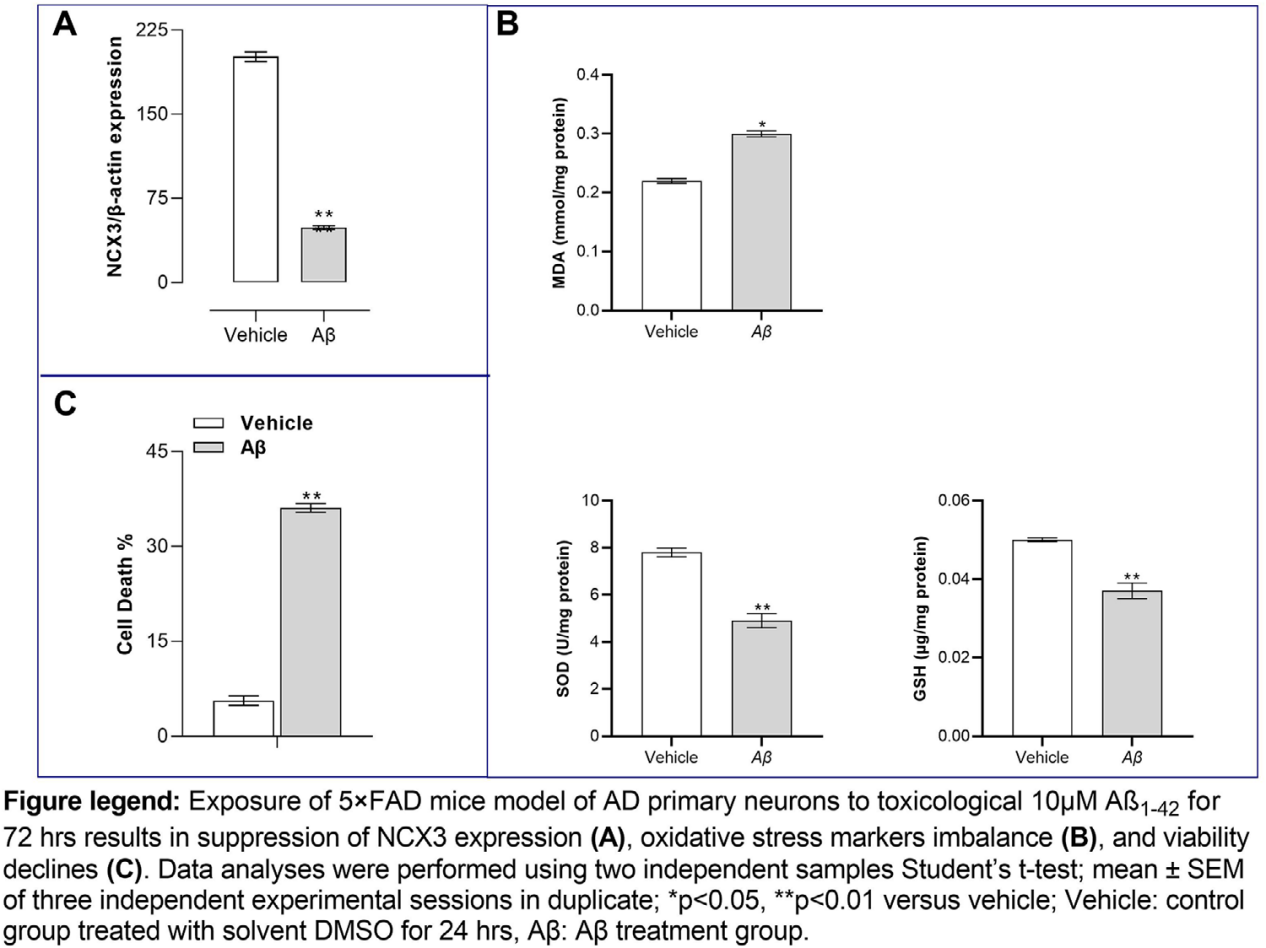# Na^+^‐Ca^2+^ exchanger isoform‐3 in Aβ_1‐42_‐induced Alzheimer’s disease pathology

**DOI:** 10.1002/alz.095787

**Published:** 2025-01-09

**Authors:** Henok Kessete Afewerky

**Affiliations:** ^1^ Schulich School of Medicine and Dentistry, University of Western Ontario, London, ON Canada

## Abstract

**Background:**

The relevance of a plasma membrane Na^+^‐Ca^2+^ exchanger isoform‐3 protein, NCX3, is widely evidenced in neuronal physiology. However, the mechanisms leading to NCX3 expression deficits in Alzheimer’s disease (AD) pathology and its value as a target in AD pharmacological medicine remain incomplete.

**Methods:**

Inhibition and rescue experiments were performed in cultured primary neurons of 5×FAD mice model of AD using pathological Aβ_1‐42_ isolated from a conditioned medium of BHK cells, a cell line which does not constitutively express NCX3, stably transfected with a plasmid expressing human wild type Aβ_1‐42_.

**Results:**

Pathological forms of Aβ_1‐42_ interfere with NCX3 expression level and neuronal viability. Pronounced expression levels of NCX3 and state of endurance were observed in the control group compared with those treated with pathological Aβ_1‐42_.

**Conclusion:**

NCX3 merits in‐depth study consideration as a predictive biomarker and in developing a potential novel pharmaceutical intervention method for AD.

**Keywords**: Alzheimer’s disease, Aβ_1‐42_, Na^+^‐Ca^2+^ exchanger isoform‐3